# Can the Digital Economy Promote the Upgrading of Urban Environmental Quality?

**DOI:** 10.3390/ijerph20032243

**Published:** 2023-01-27

**Authors:** Senhua Huang, Feng Han, Lingming Chen

**Affiliations:** 1 Department of Economics, School of Business, Hunan University of Science and Technology, Xiangtan 411201, China; 2 Department of International Economics and Trade, School of Economics and Management, Shaoyang University, Shaoyang 422000, China; 3 Department of Economics, School of Economics, Nanjing Audit University, Nanjing 211815, China; 4 Department of Economics and Statistics, School of Economics and Management, Xinyu University, Xinyu 338004, China

**Keywords:** digital economy, urban environmental quality, endogeneity, entropy method, spatial Dubin model

## Abstract

As the core of economic development, the digital economy plays an essential role in promoting urban environmental quality. In this study, we constructed a comprehensive indicator system using two dimensions, i.e., the internet and digital finance, to measure the development situation of the urban digital economy, and we used principal component analysis to assess it. From the three perspectives of ecological environment state, ecological environment pollution degree, and ecological environment governance ability, the entropy method was used to measure the quality of the urban environment. On the basis of panel data from 275 cities (prefecture-level and above) in China from 2011 to 2019, we empirically analyzed the impact of the digital economy on urban environmental quality using the two-way fixed effect model and spatial Dubin model. The research shows that the digital economy significantly promotes urban environmental quality upgrades. This conclusion still holds when considering endogeneity. This effect is mainly achieved by promoting technological innovation, optimizing the industrial structure, and enhancing market competition. Further research demonstrated that the digital economy does not significantly impact the improvement of environmental quality in small- and medium-sized cities, but has a positive effect on environmental quality upgrading in large cities. The development of the digital economy promoted urban environmental quality upgrading in the region. However, the development of the digital economy has no significant impact on environmental quality upgrading in surrounding areas.

## 1. Introduction

China is the largest developing country in the world, and the speed of its development is extraordinary. This was particularly apparent as it rapidly grew to become the world’s second-largest economy and the world’s largest manufacturing nation. This rapid industrialization drove the growth of cities. However, at the same time, as a result of increasingly severe environmental problems such as air pollution, arable land decreased [1], industrial pollution intensified [2], and carbon emissions exceeded the standard [3,4]. According to the Yale University Environmental Performance Index report in 2021, China ranked 118th, which is in a middle- to lower-position. According to the “B.P.” Statistical Yearbook of World Energy 2021, China’s carbon dioxide emissions in 2020 were 9899.3 million tons, ranking first globally. China has become the world’s largest energy consumer since 2010 [5]. Moreover, energy efficiency in China lags behind OECD countries and developing countries such as India, Mexico, and Brazil in terms of energy efficiency [6]. With global climate change and constraints related to resources and the environment, the contradiction between China’s economic growth, its energy consumption, and environmental protection are becoming increasingly serious. Climate deterioration affects the sustainable development of society [7]. Thus, improving the quality of the ecological environment and promoting sustainable development have become inevitable for China’s future development. The current industrial scenario is characterized by prominent structural contradictions, overcapacity, and large energy consumption. Thus, improving the quality of the ecological environment and the level of economic development is central to China’s future.

With the wide application of new-generation information technology (artificial intelligence, cloud computing, big data, etc.), a new economic form—the “digital economy”, supported by internet technology, artificial intelligence, and big data—has emerged. The transformation of the mode of production driven by big data and cloud computing, innovations related to modes of transaction led by digital finance and platform trading, and the networked circulation of production factors are all concrete manifestations of digital transformation [8]. The digital transformation of the global economy and society is accelerating. In addition, the digital economy is a new engine driving China’s economy and a new driving force for high-quality economic development [9]. China’s digital economy has developed rapidly in recent years. According to a white paper on the Development of China’s Digital Economy 2021, China’s digital economy represented 39.2 trillion yuan in 2020, accounting for 38.6% of GDP, and the growth rate was more than three times the nominal GDP growth rate in the same period. Moreover, digital industrialization represented 7.5 trillion yuan, a year-on-year increase of 5.3%, and industrial digitalization represented 31.7 trillion yuan, a year-on-year increase of 10.3%. At the end of 2019, the total digital economic output of 47 major economies was 31.8 trillion U.S. dollars, with an annual growth rate of 5.4%, which is higher than the global GDP growth rate of 3.1% [10], representing an essential contribution to global economic growth [11]. Therefore, the digital economy is a new driving force, which plays an essential role in stable economic growth. In this study, we explored the effect of the digital economy on urban environmental quality upgrading, assessing which mechanisms are at work and which digital economy spatial spillover effects are involved in urban environmental quality upgrading. According to the Chinese government’s 2022 work report, China will continue to focus on improving the ecological environment, and promoting green and low-carbon development, and the harmonious coexistence of man and nature. The Fourteenth Five-Year Plan of the People’s Republic of China emphasizes that we should accelerate the construction of the digital economy, promote the deep integration of digital technology and the real economy, enable the transformation and upgrading of traditional industries, and strengthen the new engine of economic development. Therefore, in this context, exploring the above issues is not only an extension of the research on the digital economy but also of great significance for the realization of the goal of “building a beautiful China”.

The existing research on the digital economy mainly focuses on the following two aspects:

First, the scale of the digital economy is assessed, or the scale or development level of the digital economy is measured. Strassner and Nicholson (2020) [12] elaborated on how the US Bureau of Economic Analysis (BEA) defines the digital economy, and how to quantify the output value of the digital economy. García and Xu (2018) [13] estimated the size of China’s digital economy based on the value added by China’s ICT industry and the number of employees. They compared it with OECD countries and believed that China’s current digital economy level had not reached the average level of OECD countries. Chinoracky and Corejova (2021) [14] built a scale evaluation system for the digital economy from three primary dimensions: economy, labor force, and technical capacity. They measured the scale of the digital economy in 19 European countries, including Belgium, from 2008 to 2018 using the entropy method. 

Second, existing research mainly focuses on the impact analysis of the digital economy as related to the speed of development. Jiao and Sun (2021) [15] conducted an empirical study on the role of the digital economy in major cities through panel data from 173 cities in China. The study found that the digital economy has a significant role in promoting economic development in China. Regarding optimizing and upgrading the industrial structure [16], Su et al. (2021) [17] used provincial level data from China to conduct an empirical study on the effect of the digital economy on industrial structure upgrading. They found that the digital economy has a positive role in promoting the improvement of industrial structures. In regards to technological innovation, Wang and Cen (2022) [18] used the spatial measurement method to conduct an empirical analysis of the role of the digital economy on innovation efficiency. The study found that the digital economy improved the innovation efficiency of the region and neighboring regions. In regards to energy, Shahbaz et al. (2022) [19] used panel data from 72 countries from 2003 to 2019 to discuss the role of the digital economy in energy. Guo et al. (2022) [20] found that smart city construction can affect regional energy by improving energy efficiency. Concerning environmental pollution, through an empirical study on the role of the digital economy in environmental haze, Li et al. (2021) [21] found that the digital economy alleviated haze pollution. The research of Xu et al. (2022) [22] shows that the digital economy can effectively reduce urban pollution. Li and Wang (2022) [8] conducted an empirical study on the carbon emission effect of the digital economy in Chinese cities. The study found that there is an inverted U-shaped relationship between the digital economy and carbon dioxide emissions in cities; i.e., the digital economy first increased carbon dioxide emissions and then reduced carbon emissions. Liu et al. (2022) [23] researched the impact of digital economy development on carbon emission efficiency based on Chinese 30 provinces and cities. Yan et al. (2020) [24] tested the heterogeneous relationship between the PM 2.5 level and the economy using two-step quantile panel regression. Yang and Ma (2021) [25] found that there is a U-shaped relationship between economic development and environmental quality using the environmental Kuznets curve. 

Climate change has become one of the world’s most serious challenges, with countries having to explore feasible ways to achieve low-carbon development [26]. Improving urban environmental quality is an inherent requirement for China to achieve sustainable development. In the existing literature, few studies directly discuss the impact of the digital economy on urban environmental quality upgrading. This paper approaches the subject from two aspects: theory and an empirical analysis of the influence of the digital economy on upgrading the quality of the urban environment. The main contributions of this paper are as follows: First, previous studies on environmental pollution mainly analyzed a single indicator of environmental pollution, or used a comprehensive index of pollution emissions. There are few studies that conducted a thorough evaluation of environmental quality at the urban level, and it is rare to directly discuss the impact of the digital economy on urban environmental quality upgrading. On the basis of the comprehensive evaluation of urban environmental quality, this paper identifies the mechanisms at work. We conducted empirical tests, which represent an expansion of the existing research on the theme of digital economy and environmental pollution. Second, this paper examines the spatial spillover effect of the digital economy on urban ecological quality upgrading and explores the heterogeneity characteristics of the digital economy on urban environmental quality upgrading from the perspective of different city sizes. This allowed us to conduct a deeper study on the impact of the digital economy on urban ecological quality upgrading.

The remainder of this paper is arranged as follows: The second part is made up of the theoretical analysis and research hypothesis, which provide a solid theoretical foundation for this study. The digital economy promotes urban environmental quality upgrading through technological innovation, optimizing the industrial structure, and promoting market competition. The third part comprises the methodology and econometric model, and includes details of the variable measurements and data description. The fourth part presents the empirical test and results from the analysis, which includes the total sample estimation, robustness test, and mechanism of action test. The fifth section is the discussion based on different city sizes and spatial spillover effects. The sixth part presents the research conclusions and policy recommendations.

## 2. Theoretical Analysis and Research Hypothesis

### 2.1. Digital Economy through Technological Innovation to Promote Urban Environment Quality Upgrading

Digitalization has become a new economic form. The ICT industry in the field of the digital economy itself is a knowledge-intensive industry with an abundance of innovation resources, frequent internal innovation activities, and constant digital innovation achievements [27]. Firstly, the application of digital technology has greatly improved the intellectual level of production equipment. Real-time information collection and feedback can be achieved by introducing production equipment with computing communication, precise control, remote coordination, self-management, and other functions. It is helpful to optimize the production process according to production dynamics and to promote the collaboration on the production line [28].

Along with this process, many enterprises gradually shift from resource-intensive manufacturing to technology-intensive manufacturing [29], thereby improving production efficiency. The application of digital technology promotes the research and development of efficient equipment and replaces low-energy production equipment with high-energy production equipment, undoubtedly improving production efficiency. Secondly, the development of the digital economy is conducive to accelerating the cross-regional integration of innovation resources. Innovation elements such as capital, talent, and technology in different regions can be effectively integrated under the double effect of network link points [10] and the reorganization of these innovation elements. According to Schumpeter’s innovation theory, reorganizing different production factors is an important part of innovation [30]. The effective integration of fragmented R&D resources and information knowledge has improved technological innovation. Thirdly, technology communication within the whole society has been promoted by the digital economy [31]. Using the internet, artificial intelligence, and other digital technologies, it is more convenient for all innovation subjects to obtain information and knowledge. The flow efficiency of expertise has been significantly improved among regional innovation systems, promoting the spread of new technologies and expertise. Therefore, the digital economy promotes the construction of an intelligent, green, and low-carbon industrial manufacturing system, supports technological innovation, and thus promotes the upgrading of urban environmental quality. Therefore, we propose the following hypothesis:

**Hypothesis 1:**  *The digital economy promotes urban environmental quality upgrading by promoting technological innovation.*

### 2.2. Digital Economy Promotes Urban Environmental Quality Upgrading by Optimizing Industrial Structure

Upgrading the industrial structure is a complex process: the original resources are re-allocated from low-end to high-allocation industries, and new resources flow to high-tech and high-tech-intensive industries so that the proportion of technology-intensive industries continues to rise [32]. The digital economy is a new economic and social operation based on information and communication technology [33]. The digital economy is regarded as a technological revolution that will have a huge impact on all forms of industry [34]. Digitization and information are an important part of the digital economy. They broaden the boundaries of the division of labor in the industrial chain and accelerate the transformation of traditional industries, especially manufacturing. In the integration of the digital economy and conventional industries, the intelligence and digitalization of enterprises are promoted. Thus, the industrial structure develops from traditional labor and capital-intensive industries to data-intensive and technology-intensive industries [10]. Simultaneously, a digital economy with high innovation, strong penetration, and rapid diffusion is also conducive to promoting exchanges and cooperation between upstream and downstream enterprises and promoting the integrated development of the industry [35,36]. The integrated development of related industries brings into play their comparative superiority, promoting horizontal and vertical cooperation, thereby forming economies of scale and regional economies, thus pushing the whole industrial chain to a higher value. Therefore, as digital economies progress, the proportion of technology- and digital-related industries increases, which promotes the optimization and improvement of the industrial structure. Thus, the efficiency of industrial production is greatly improved, achieving the goals of low input, high output, and low pollution. In this way, the entire industrial system develops in a green direction, thereby improving the ecological environment of the city. Therefore, the following hypothesis is proposed in this article:

**Hypothesis 2:**  *The digital economy promotes urban environmental quality upgrading by promoting industrial structure optimization.*

### 2.3. Digital Economy Promotes Urban Environmental Quality Upgrading by Promoting Market Competition 

The wide application of information technologies such as the internet and big data has broken the information gap in the market, making the market more transparent and competitive [37]. Digitalization has led to an increased amount of business models. The deep integration of digital technology and the real economy has subverted and reshaped the value creation model of traditional industries. The digitalization process can accelerate the integration of information resources, lower barriers to entry for new businesses, and help avoid monopolies [38]. Because digitalization is convenient, the digital economy is also characterized by convenient replication and dissemination. Of course, different industries have different barriers to entry. In the era of the digital economy, the application of digital technology promotes the digital transformation of elements. With the help of digital technology, such as internet technology, consumers and suppliers of features can defy time and space constraints, achieve effective communication and feedback, and reduce the search cost of components. Therefore, the digital economy reduces the market entry threshold on the supply side and the information retrieval cost and comparison cost of consumers on the demand side, enhancing market competition. Additionally, the rapid progress of online digital trading platforms has, to a certain extent, obscured regional restrictions on business operations and intensified competition between enterprises in different regions [39]. In the current context of paying more attention to the ecological environment, enterprises must consider economic and environmental benefits to achieve long-term development. On the one hand, the government should strengthen the revision and improvement of environmental laws and regulations, and conditionally implement special subsidy policies. Moreover, it also forces enterprises to internalize reasonable environmental costs. On the other hand, enterprises should also implement green quality management and incorporate green measures into daily production and operation management. The strengthening of market competition will force enterprises to increase the use of green production equipment, transform production processes, improve resource utilization efficiency, promote green production, and, thus, promote urban environmental quality upgrading. Therefore, the following hypothesis is proposed in this paper:

**Hypothesis 3:**  *The digital economy promotes urban environmental quality upgrading by enhancing market competition.*

## 3. Methodology and Econometric Model

### 3.1. Economic Model Construction

To evaluate the influence of the digital economy (DE) on urban environmental quality (EQ) and to minimize the estimation bias caused by the omission of other variables, drawing on the research of Ma et al. (2022) [11], Han et al. (2021) [40], and Wang et al. (2022) [41], the agglomeration of producer services *(*sagglo), foreign direct investment (lnfdi), economic development level (lnpgdp), human capital (H), and scientific and technological expenditure (rd) were selected as important factors affecting urban environmental quality. Therefore, they were taken as control variables, and the non-proportional variables were logarithmized. The measurement model was constructed as follows:

(1) $$\ln EQ_{it}=\alpha_{0}+\alpha_{1}\ln DE_{it}+\alpha_{2}\ln sagglo_{it}+\alpha_{3}\ln fdi_{it}+\alpha_{4}\ln pgdp_{it}+\alpha_{5}\ln H_{it}\;+\alpha_{6}rd_{it}+\mu_{i}+\nu_{t}+\varepsilon_{it}$$

where i and t denote city and year, respectively, α represents the parameters to be estimated, $\mu_{i}$ and, $V_{t}$ represent the urban and time-fixed effects, respectively, and ε represents a random perturbation term.

In light of the above action mechanism analysis, the urban environmental quality can be promoted by the digital economy through promoting technological innovation, optimizing the industrial structure, and improving the market environment. To verify the role of these mechanisms, we used the work of Ye and Zhuang (2022) [42] as a reference to build the following test model:

(2) $$\ln M_{it}=\beta_{0}+\beta_{1}\ln DE_{it}+\phi_{i}Control_{it}+\psi_{i}+\kappa_{t}+\eta_{it}$$

where M is an intermediate variable, Conrol is a control variable, i and t denote the city and year, respectively, β denotes the parameters to be estimated, $\psi_{i}$ and $K_{t}$ represent the fixed effects of city and time, respectively, and η represents the random disturbance term.

### 3.2. Variable Measurement and Data Description

The research sample in this paper is made up of data from 275 cities in China from 2011 to 2019 (not including cities under the jurisdiction of Tibet, Hong Kong, Macao, and Taiwan). The data are from the Statistical Yearbook of Chinese Cities, the Statistical Yearbook of China’s Regional Economy, the Digital Inclusive Financial Index of Peking University (2011-2019), the Statistical Yearbook of China, the China Research Data Service Platform (CNDRS), the statistical yearbooks of provinces and cities, and the Bulletin on Urban National Economic Development and Social Statistics. Carbon emission data were taken from the county carbon dioxide emissions calculated by Chen et al. (2020) [43], summarized at the city level in 2017. The quadratic exponential smoothing method was used to estimate the carbon emission data from 2018 to 2019. Descriptive statistics of variables are shown in Table 1. The detailed indicator measures are as follows:

#### 3.2.1. Explained Variable

Urban environmental quality (EQ). The quality of the ecological environment reflects the suitability of the ecological environment for human survival and sustainable socio-economic development [44]. The ecological environment quality comprehensively reflects the natural state, environmental pollution, governance level, and other specific human requirements [45,46]. Therefore, urban ecological quality cannot be reflected by a single indicator but needs a comprehensive indicator system for measurement. Drawing on the research of Han et al. (2021) [40] and considering the availability and unity of urban-level data, we constructed a comprehensive indicator system of urban environmental quality from three dimensions: “ecological environment state”, “ecological environment pollution degree”, and “ecological environment governance capacity”. Table 1 shows the breakdown indicators. As in the research of Chen et al. (2021) [44], the entropy method was used to measure urban environmental quality. Both positive and negative indicators needed to be normalized. For that specific process, the practices of Wang et al. (2021) [47] and Zhao et al. (2018) [48] were used as a reference. Equation (3) was used for the positive indicators, and Equation (4) was used for the negative indicators. The specific processing formula is as follows:

(3) $$x_{i,j}=\frac{x_{i,j}-\min\{x_{j}\}}{\max\{x_{j}\}-\min\{x_{j}\}}$$

(4) $$x_{i,j}=\frac{\max\{x_{j}\}-x_{i,j}}{\max\{x_{j}\}-\min\{x_{j}\}}$$

where $max\{x_{i,j}\}$ is the maximum value of indicators in all years, $min\{x_{j}\}$ is the minimum value of indicators in all years, and $x_{i,j}$ is the dimensionless result. The weight of the *j* indicator in year *i* was calculated, and is expressed as $\omega_{i,j}$*:*

(5) $$\omega_{i,j}=\frac{x_{i,j}}{\sum_{i=1}^{m}x_{i,j}}$$

The information entropy $e_{j}$ of the indicator was defined. Therefore,

(6) $$e_{j}=-\frac{1}{\ln m}\sum_{i=1}^{m}\omega_{i,j}\times \ln\omega_{i,j}$$

Information entropy redundancy $d_{j}$ was calculated:

(7) $$d_{j}=1-e_{j}$$

In this, m is the evaluation year, and the index weight $\varphi_{j}$ was calculated according to the information entropy redundancy:

(8) $$\varphi_{j}=\frac{d_{j}}{\sum_{j=1}^{m}d_{j}}$$

On the basis of index $x_{i,j}$ and weight $\varphi_{j}$, the index level (EQ) of urban environmental quality was calculated. The calculation formula is as follows:

(9) $$EQ_{j}=\sum_{j=1}^{m}\varphi_{j}\times \omega_{i,j}$$

where $EQ_{j}$ represents the comprehensive indicator of urban environmental quality in urban area i, which is between 0 and 1. The larger $EQ_{j}$, the higher the urban environmental quality. On the contrary, the smaller $EQ_{j}$, the lower the urban environmental quality.

#### 3.2.2. Core Explanatory Variable

Digital economy (DE). Currently, there is no unified standard for the connotation and measurement of the digital economy. To define and measure the digital economy, in the existing research, many scholars define and measure it using internet development and digital finance [8,21,49,50]. Therefore, in this paper, we estimated the development level of the urban digital economy from two aspects: internet development and digital finance, including five indicators: internet penetration rate, mobile phone penetration rate, internet-related practitioners, internet-related industrial output, and digital finance development. See Table 2 for details. This paper uses the principal component analysis method to measure the digital economy development index. The principal component analysis method was used to reduce the dimension of the comprehensive index system. Since the Stata software has no panel processing commands, processing was performed in sections; i.e., individually for each year. Five secondary indexes of digital economy are used in this paper. In this study, we first eliminated the dimensional differences of these variables. Secondly, the Bartlett sphericity test and KMO test were performed on these variables, and the test results supported the factor analysis. This also indicates that there is a strong correlation between the indicators, and the original data meet the conditions for principal component analysis. Each of the principal components is a linear combination of the original variables, and the individual principal components are independent from each other. The principal components were extracted according to the size of the eigenvalues and the cumulative contribution rate of variance [51]. By means of principal component analysis, the data of the above five indicators were standardized and processed with dimensionality reduction, and the obtained comprehensive development index of digital economy is denoted as DE.

#### 3.2.3. Control Variables 

The producer services agglomeration (sagglo), referring to Zhao et al. (2021) [52], measures the agglomeration level of producer services in various regions by the location entropy index, and the specific formula is as follows:

(10) $$sagglo_{ij}(t)=\frac{e_{ij}(t)/\sum_{i}e_{ij}(t)}{\sum_{j}e_{ij}(t)/\sum_{i}\sum_{j}e_{ij}(t)}$$

where $sagglo_{ij}\left(t\right)$ represents the agglomeration level of j industries in i regions in t periods, $e_{ij}\left(t\right)$ represents the number of employees in j industries in i regions in t periods, $\sum_{i}e_{ij}(t)$ represents the number of employees in j industries in China in t periods, $\sum_{j}e_{ij}(t)$ represents the number of employees in all industries in i areas in t periods, and $\sum_{i}\sum_{j}e_{ij}(t)$ represents the number of employees in all industries in China in t periods. For the selection of producer services, drawing on the work of Xie et al. (2019) [53], in this study, we selected the following consolidated data from seven industries to calculate the concentration degree of producer services: the transportation, warehousing, and postal industry; the wholesale and retail industry; the financial industry; the leasing and commercial service industry; the information transmission, computer service, and software industry; the scientific research and technical service industry; and the environmental governance and public facility management industry.

Foreign investment (fdi) is expressed by the total foreign direct investment actually used by the city. This was converted into the fixed price in 2003 based on the CPI index at the provincial level. The level of economic development (pgdp) is expressed in terms of the per capita GDP of the city and was converted into the constant price in 2003 using the provincial GDP deflator. Human capital (H) is expressed by the quantity of students in higher learning institutions. The proportion of science and technology expenditure in the total financial expense in the budget represents science and technology expenditure (rd).

#### 3.2.4. Mediating Variables

The number of urban patent authorizations represents technological innovation (PT). Industrial structure optimization (S) is described by the ratio of the output value of the tertiary industry to that of the secondary industry. Using the work of Liu and Gu (2015) [54] as a reference, the average wage of urban employees was used as a measure of market competition (MC), which was converted into the constant price in 2003 based on the CPI index at the provincial level, Table 3.

### 3.3. Measurement Results of Environmental Quality and Digital Economy

#### 3.3.1. Measurement Results of Environmental Quality

**Figure 1:**
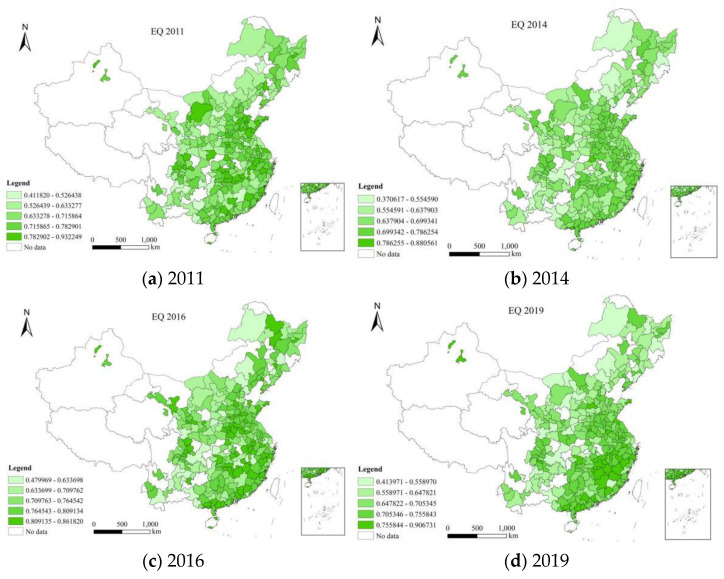
Environmental quality of 275 cities in China in (**a**) 2011, (**b**) 2014, (**c**) 2016 and (**d**) 2019.

This paper uses the data from 2011–2019 to measure the environmental quality of 275 cities in China using entropy method. ArcGIS 10.8 software (Environmental Systems Research Institute (Esri), RedLands, CA, USA) is used to draw the map. The darker the color is, the better the level of environmental quality is. Figure 1 shows the environmental quality level of 275 cities in China in 2011, 2014, 2016 and 2019.The development level of environmental quality in different regions of China showed different trends from 2011 to 2019. Specifically, it can be divided into the following situations: First, Shenzhen, Suizhou, Suining and Huangshan ranked first in environmental quality in 2011, 2014, 2016 and 2019; Second, the top five cities of environmental quality in 2011 are Shenzhen, Shiyan, Ordos, Suizhou and Karamay;Third, the top five cities of environmental quality in 2014 were Suizhou, Beihai, Shenzhen, Huangshan and Karamay; Fourth, the top five cities of environmental quality in 2016 were Heihe, Sanya, Zhuhai, Luohe, Suining; Fifth, the top five cities of environmental quality in 2019 are Huangshan, Zhoushan, Karamay, Shenzhen and Jingdezhen. Shenzhen is a pioneer city in China’s economic reform, focusing on economic growth. However, it also pays more attention to environmental quality. In general, cities that focus on environmental quality may have slower economic growth. This is mainly because the improvement of environmental quality requires certain economic costs.

#### 3.3.2. Measurement Results of Digital Economy 

**Figure 2:**
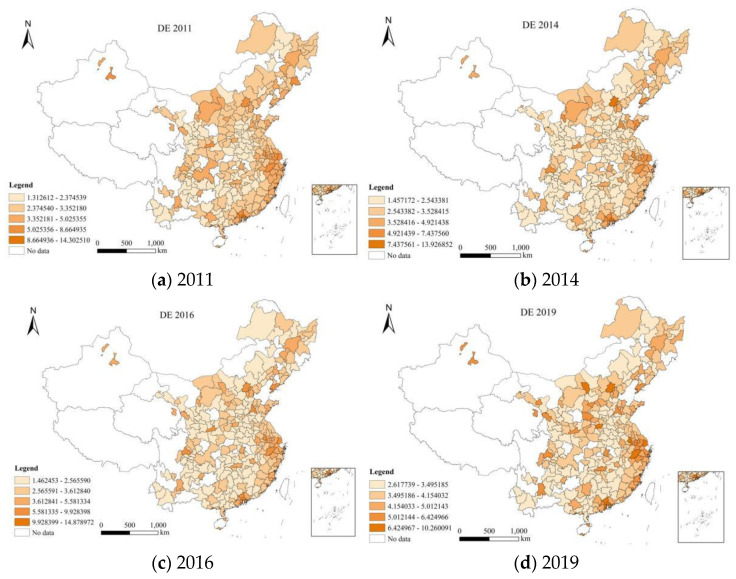
Digital economy of 275 cities in China in (**a**) 2011, (**b**) 2014, (**c**) 2016 and (**d**) 2019.

This paper uses the data during the year of 2011–2019 to measure the digital economy of 275 cities in China using principal component method. ArcGIS 10.8 software is used to draw the map. The darker the color is, the better the level of digital economy is. Figure 2 shows the digital economy level of 275 cities in China in 2011, 2014, 2016 and 2019.The development level of digital economy in different regions of China showed different trends during the year of 2011 to 2019. Specifically, it can be divided into the following situations: First, Shenzhen, Shenzhen, Shenzhen and Zhuhai ranked first in the development level of digital economy in 2011, 2014, 2016 and 2019;Second, the top five cities of digital economy in 2011 are Shenzhen, Dongguan, Beijing, Guangzhou and Zhongshan; Third, the top ten cities in the digital economy in 2014, 2016 and 2019 are basically the same. The development level of digital economy is mainly concentrated in large cities and coastal cities. The main reason may be that the digital economy infrastructure in these cities is good. Compared with other cities, the digital economy develops more rapidly.

## 4. Empirical Test and Result Analysis

### 4.1. Full Sample Estimation Results

In order to overcome the influence of non-observational factors, to reduce endogenous errors as much as possible, and to improve the reliability of measurements, we used a two-way fixed effect model to study the impact of city and time. Moreover, only fixed urban impacts are listed for comparison. Table 4 shows the results of the estimation of the impact of the digital economy on urban environmental quality. The estimated results listed in Column (1) of Table 4 are the results without the addition of the control variables and controlling for time fixed effects, while in Column (2), there is no control variable, but time and urban effects are all controlled. In addition, after introducing the control variable, the third column is the non-controlling time effect, and the fourth column is the controlling time and urban influence. From the results in Column (2) and (4), in terms of controlling time and urban effect, the estimated coefficient of the digital economy is significant at approximately 5%, which shows that the development of the digital economy played a positive role in improving the environmental quality in cities. This is mainly because, with the rapid growth of the digital economy, the application of network technology in the field of environmental protection is becoming increasingly extensive, thus, the level of emission reduction technology has improved [55,56]. On the other hand, the digital economy provides support for low-carbon technology enterprises and creates conditions for the technological development of related industries [57].

The following section focuses on the estimation results of the control variables. Although the estimated coefficient of science and technology expenditure (rd) was positive, it failed the significance test, indicating that science and technology expenditure did not have a significant impact on urban environmental quality, which may show that the government’s science and technology expenditure on environmental pollution control was lacking, resulting in insufficient support for green technology R&D activities. Thus, the promotion effect of science and technology expenditure on environmental quality upgrading was not clear to see. The estimated coefficient of human capital (lnH) was insignificant in the negative direction, which may show that, although the number of people receiving education in China is increasing, the quality of education has not improved with the increase in the number of those in education. There are still more people engaged in low-tech industries, which is unfavorable to the progress of green technology, thus, leading to the failure of human capital to significantly promote urban environmental quality. The estimated coefficient of producer services agglomeration (lnsagglo) was negatively insignificant. This means that producer services agglomeration restrained the improvement of urban environmental quality, which may be related to the insufficient development of high-end producer services in China. The blind development of producer services by local governments under the promotion of “retreat from two to three” industrial planning affects the scale effect and technology spillover effect of producer services agglomeration. This results in the promotion effect of producer services agglomeration on urban environmental quality upgrading not being visible. Moreover, foreign direct investment (lnfdi) has no significant impact on urban environmental quality. 

On the one hand, the introduction of foreign capital brings clean production technology to the host country and promotes green development, on the other, it may also bring about the transfer of polluting industries, thus restricting the improvement of urban environmental quality. In terms of positive and negative effects, it was shown that foreign investment did not have a significant impact on the urban environmental quality in the investment area. The estimated coefficient of economic development level (lnpgdp) was positive and statistically significant, which shows that economic development promoted urban environmental quality upgrading.

### 4.2. Robustness Test

Although in this study, we controlled for the factors affecting urban environmental quality, there may be other potential factors affecting urban ecological quality. It was a challenge to include these in the model construction. The elements not considered are included in the random disturbance term, which can lead to endogenous problems. To address this, we adopted 2SLS for estimation, i.e., the lag period I and II of the digital economy were selected as instrumental variables. In addition, in the China “internet +” index report (2015–2019), the Tencent Research Institute proposed “internet +” as an alternative index and used a new impact model to analyze the previous data to make a prediction. The 2019 “internet +” index did not provide the “internet +” in 2019, and it was estimated by multiplying the average growth rate from 2015 to 2018 by the 2018 “internet +” index. 

Table 5 shows these results. Column (1) was used to estimate the forecast result of the first and second phases of the digital economy development, and Column (2) shows the “internet +” digital economy index, which was used as an alternative variable for evaluating the development degree of the digital economy. The results of K.P. rk L.M. and KP rk Wald F in Column (1) of Table 5 show that the instrumental variables are reasonable and effective. That is to say that there, the study did not include any insufficiently identified instrumental variables or weak instrumental variables. In the case of possible inherent problems and alternative measurement variables, the estimated coefficient of the digital economy was at least 10%, and the digital economy’s development had a significantly positive effect on economic development. The environmental quality in cities shows that the digital economy’s development promoted the environmental quality in cities, which verifies the regression results of previous econometrics.

### 4.3. Mechanism of Action Test

Table 4 and Table 5 show the results confirming that the digital economy promotes the improvement of urban environmental quality. However, its transmission path still needs to be analyzed. Table 6 shows the results of the mechanism test based on Model (2). Among them, Column (1) shows the test result of the technological innovation mechanism, Column (2) shows the test result of the industrial structure optimization mechanism, and Column (3) shows the test result of the market competition mechanism. From the results in Column 1, we can see that the direction of the estimated coefficient of the digital economy (lnDE) is positive and passes the significance test at the 1% level. This demonstrates that the digital economy promoted technological innovation, which verifies theoretical hypothesis 1, i.e., digital economy → technological innovation → urban environmental quality upgrading. The development of the digital economy brought about the application of digital technology, efficiently integrated innovative resources, promoted the spread of new technologies and knowledge, enhanced technological innovation, promoted green and low-carbon development, and improved the quality of the urban environment. From the results of Column (2) estimation, the development of the digital economy played a conspicuous role in promoting the optimization of the industrial structure, which confirms hypothesis 2. In the integration of the digital economy and conventional industries, the high permeability and strong diffusion characteristics of the digital economy promote intelligence and the digitalization of industry. Thus, the industrial structure shifts to a data-intensive and technology-intensive mode, and the middle-to-high-end development of the industrial structure is achieved. In this manner, the industrial structure is optimized, and the quality of industry and the urban environment is improved. It can be seen from the third column that the estimated coefficient of the digital economy is significantly positive at 1%, which shows that the digital economy can promote market competition and confirms the previous theoretical assumption; i.e., the development of the digital economy affects the supply and demand of various factors, defying the boundaries of time and space, reducing information asymmetry, and improving market transparency. The development of a network digital trading platform breaks the localization of the commodity trading market. Furthermore, it promotes market competition and forces enterprises to improve resource utilization efficiency and green production, thus, urban environmental quality upgrading is realized.

## 5. Discussion

### 5.1. Analysis Based on Different City Sizes

Considering that cities of different sizes have different resource endowments, internet infrastructure investment, and industrial development levels, the impact of the digital economy in various cities on urban environmental quality may be heterogeneous. For this reason, according to the Notice on Adjusting the Standards for the Classification of City Size issued by the State Council, cities were divided into two categories: large cities (with a population of more than 1 million), medium and small cities (with a population of 1 million and below), according to the population of the municipal districts at the end of the year. See Table 7 for specific cities.

The estimated results from the perspective of different city sizes are shown in Table 8. The estimation results show that in the sample of large cities, the estimation coefficient of the digital economy was significantly positive, indicating that the digital economy promoted environmental quality upgrading in large cities. In the sample of medium and small cities, although the estimation coefficient of the digital economy was positive, it failed to pass the significance level test which indicates that the digital economy did not have a significant impact on environmental quality upgrading in medium and small cities. The reason for this may be that, as compared with medium and small cities, the digital economy in big cities develops earlier and at a higher level, which is conducive to the full realization of the advantages associated with the digital economy. Thus, urban environmental quality upgrading is promoted. The development of the digital economy in medium and small cities appeared to be lagging in comparison, and its role in promoting urban environmental quality upgrading could not be seen.

### 5.2. Analysis of Spatial Spillover Effect

There is spatial autocorrelation in urban environmental quality (Han et al., 2021) [40]. Moreover, the typical cross-regional division of labor and collaboration in developing a digital economy means that a region’s environmental quality is affected by adjacent areas’ environmental quality and the neighboring regions’ digital economy. Moreover, combining LeSage and Pace (2009) [58] to carry out the unbiased estimation using the spatial Dubin model, we constructed the following spatial Dubin model for the spatial econometrical analysis:

(11) $$\ln EQ_{it}=\gamma_{0}+\rho W\ln EQ_{jt}+\gamma_{1}\ln DE_{it}+\gamma_{2}W\ln DE_{jt}+\kappa_{i}X_{it}+\chi_{i}WX_{jt}+\delta_{i}+\lambda_{t}+\sigma_{it}$$

where ρ denotes the spatial lag coefficient, X represents a vector of control variables, $\gamma_{1}$, $\gamma_{2}$, $K_{i}$, and $X_{i}$ are the elastic coefficients of the variable, $\delta_{i}$ and $\lambda_{t}$ represent the time effect and space effect, respectively, $\sigma_{it}$ is the random disturbance term, and W represents the spatial weight matrix. On the basis of the research of Zhang et al. (2021) [59], we constructed three matrices: the geographic distance matrix ($W_{1}$), the economic distance matrix ($W_{2}$), and the economic and geographic nested matrix ($W_{3}$). Among them, $W_{1}$ was constructed with the reciprocal surface distance between cities, as measured using latitude and longitude, and the economic distance matrix $W_{2}=1/\left|\bar{Q_{i}}-\bar{Q_{j}}\right|$, where $\bar{Q_{i}}$ and $\bar{Q_{j}}$ represent the average GDP per capita of cities i and j (i≠j) in 2011–2019, respectively. In the economic and geographic distance nested matrix, $W_{3}=\lambda W_{1}+(1-\lambda )W_{2}$, and λ takes 0.5. On the basis of the geographic distance matrix ($W_{1}$), in this paper, we use the Moran I index to test the spatial autocorrelation of urban environmental quality. The panel’s Moran’s I index value of urban environmental quality was 0.1057, which passed the statistical significance test at the 1% level, indicating that urban environmental quality had an obvious spatial correlation, i.e., it demonstrated the characteristics of agglomeration in a spatial distribution.

According to the study of LeSage and Pace (2009) [58], as a result of a large amount of interactive information in adjacent areas, there are deviations in explaining the spatial regression results solely using the regression coefficient. To this end, we learned from their study and used the partial differential changes of variables to explain this, i.e., the direct effect was used to express the influence of each variable on the environmental quality of its region, and the indirect effects were used to describe the impact of each variable on the environmental quality of its adjacent area. Table 8 shows the estimated results of the direct and indirect effects of SDM models based on three spatial weight matrices. According to Table 8, under the three spatial weight matrices, the direct effect of the digital economy on urban environmental quality was significantly positive, but the coefficient of indirect effect was negative and not significant, which demonstrates that the digital economy promotes environmental quality upgrading in its own region but does not have a significant impact on environmental quality in adjacent areas. Possible reasons for this are as follows: the spread of information across time and space brought about by the digital economy promotes the development of interregional economic activities, reduces information asymmetry, has an external effect, promotes technological progress, promotes green growth, and then promotes environmental quality upgrading in local cities and surrounding cities. However, at the same time, there are also strategic interactions and competitive behaviors between local governments. If a city has a high level of digital economy development, its surrounding cities will try to catch up and intensify efforts to promote the development of the digital economy. With the continuous increase in investment in digital infrastructure construction, large-scale construction will expand the economic scale. Thus, an energy rebound effect will be produced due to the expansion of the economic scale. Finally, energy consumption will increase. This intensifies pollutant emissions and is not conducive to upgrading environmental quality. The energy intensity of the surrounding area increased with the development of the internet (Hao and Wu, 2021) [60]. Positive and negative forces offset each other and the spatial spillover effect brought about by the digital economy fails to play an effective role. The specific results of direct and indirect effects are shown in Table 9.

## 6. Conclusions, Policy Recommendations, and Future Research

### 6.1. Conclusions

On the basis of panel data from 275 cities in China (prefecture-level and above) from 2011 to 2019, we empirically tested the impact of the digital economy on urban environmental quality upgrading using the two-way fixed effect model and spatial Dubin model. The main conclusions are as follows: 

First, in this study, we constructed a comprehensive indicator system of urban environmental quality from three dimensions: ecological environment state, ecological environment pollution degree, and ecological environment governance ability, and nine three-level indicators. The entropy method was used to measure urban environmental quality. Moreover, we constructed a comprehensive evaluation indicator system for the urban digital economy using five dimensions: internet penetration rate, mobile phone penetration rate, internet-related practitioners, internet-related industry output, and digital finance development. The principal component analysis method was used to measure the digital economy development index.

Second, from the estimation results from the full sample, when the control variable was introduced, the estimation coefficients of the digital economy were significantly positive at the level of 5%. This shows that the development of the digital economy significantly improved urban environmental quality upgrading. This conclusion was shown to still be valid after replacing the core explanatory variables and addressing the endogenous problems. 

Third, from the results of the theoretical analysis, the estimated coefficient of the digital economy was significantly positive at the level of 1%. This demonstrates that the three intermediary mechanisms were well-established. The digital economy can affect urban environmental quality upgrading by promoting technological innovation, optimizing the industrial structure, and enhancing market competition. 

Finally, from the city-scale perspective, the digital economy was shown to boost the environmental quality upgrading in big cities. However, the impact in medium and small cities was not obvious. The digital economy of a city boosted environmental quality upgrading in that city; however, the role of the digital economy in environmental quality upgrading in surrounding cities is not obvious. 

### 6.2. Policy Recommendations

On the basis of our conclusions, the following policy recommendations are put forward: 

Firstly, digital construction should be strengthened to make the digital economy a “sharp tool” for upgrading urban environmental quality. Digital infrastructure construction is the foundation and prerequisite for developing the digital economy. Therefore, the government should further increase investment in digital infrastructure, such as investing in the construction of new digital infrastructures such as 5G, the internet of things, industrial internet, artificial intelligence, and blockchain, to further strengthen the application of digital technology, promote the penetration of the digital economy in the real economy, and effectively promote digital economy development. Thus, the digital economy’s lifting effect on urban environmental quality upgrading can be brought into full play.

Secondly, relying on the three primary channels of technological innovation, industrial structure optimization, and market competition enhancement, the role of the digital economy in improving urban environmental quality should be fully leveraged. While actively encouraging enterprises to carry out technological R&D and innovation, the government should also provide financial and institutional support for R&D and innovation in enterprises, paying attention to the training of innovative digital talents and improving independent innovation ability. All of these areas should actively promote the optimization of industrial structures based on their economic development and resource endowment. In addition, we should actively create a fair competition market environment, promote benign and fair competition, and enhance the degree of market competition. 

Thirdly, big cities should fully utilize their digital economic foundations, expand the breadth of the digital industry in the essential, core, and high-end fields, establish a digital economy development platform and a digital economy service database, share data resources, form a large-scale high-value network, effectively promote radiation and the spread of large-scale, digital technology in small- and medium-sized cities, which will produce space spillover effects. For example, the proportion of 4G and optical fiber in administrative villages across China is in excess of 98%, and the IPv6 upgrade of the fixed broadband mobile LTE network is complete. The construction of a new generation of cloud computing platform facilities is accelerating and multi-directional and high-capacity international transmission network architecture has also been essentially formed. These are solid foundations for the digital economy and governments and enterprises should make full use of them. While medium and small cities are actively developing the digital economy, the government should give more policy support to accelerate the construction of the digital infrastructure, to enhance the attractiveness of cities, and to strengthen talent and technology reserves. In this manner, the benefits of the digital economy will be better realized.

Fourthly, given that the spatial spillover effect of the digital economy in terms of improving urban environmental quality is not obvious, cities with a backwards digital economy cannot blindly catch up and expand, but should gradually promote the development of the digital economy based on various scenarios; for example, economic development level, the status of internet infrastructure, industrial development, and information technology talents. Moreover, the government should actively promote digital resources in certain regions to narrow the digital gap between regions, to enhance the extent and depth of economic activities in different areas, to facilitate the inter-regional flow of production factors, and to effectively utilize the spatial externalities of the digital economy. For example, small- and medium-sized enterprises in large cities have made greater efforts to promote digital transformation. The government should also promote the popularization of digital technology and drive the economic development of backward regions. In doing this, the government should make full use of the new generation of information economy technology and digital economy scene models to build a “regional model” of “digital poverty alleviation” and increase the development of the digital economy in backward regions.

### 6.3. Future Research

As a result of the limited availability and applicability of data, this article only used data from 275 cities (prefecture-level and above) in China from 2011 to 2019. Therefore, the research in this article has certain limitations in terms of depth and breadth. The digital economy helps to improve the quality of the urban environment; however, urban environmental quality is definitely not determined by the digital economy alone. It is determined by many comprehensive factors, such as waste disposal, industrial production mode, traffic conditions, and residents’ behavior. This study only assessed the improvement of urban environmental quality from the perspective of the digital economy, and the authors will continue to focus on the improvement of urban environmental quality from these perspectives.

## Figures and Tables

**Table 1:** Urban environmental quality measurement index system.

Comprehensive Evaluation Index	Indicators by Category	Specific Measurement Indicators
Urban environmental quality	Ecological environment status	The green coverage rate of built-up area (%)
		Green area per capita (m^2^)
	Ecological environment pollution degree	Industrial wastewater discharge (10,000 tons)
		Industrial sulfur dioxide emissions (tons)
		Industrial soot emissions (tons)
		Carbon dioxide emissions (million tons)
	Ecological environment governance capacity	The comprehensive utilization rate of general industrial solid waste (%)
		Centralized sewage treatment rate (%)
		Harmless treatment rate of domestic waste (%)

**Table 2:** The evaluation index system of urban digital economy.

Primary Index	Secondary Index	Indicator Description	Indicator Attribute
Digital economy	Internet penetration rate	Number of internet users per 100 people	positive
	Mobile phone penetration rate	Number of mobile phone users per 100 people	positive
	internet-related practitioners	The proportion of employees in computer and software industries in the total number of employees	positive
	The output of internet-related industries	Telecom business volume per capita	positive
	Development of digital finance	Digital inclusive finance index	positive

**Table 3:** Statistical description of variables.

Variables	Mean	Standard Deviation	Min	Max
EQ	0.7026	0.0887	0.3685	0.9322
DE	3.3546	1.4414	1.0582	14.8790
rd	1.6659	1.6763	0.0598	20.6835
H	96,777.8600	169,371.8000	1.0000	1,152,995
sagglo	0.8119	0.3806	0.2433	13.6159
fd *i*	443,920.9000	1,050,464	1.0000	1.47 × 10^7^
pgdp	39,128.2100	39,181.8900	4304.3310	372,234.5000

**Table 4:** Full sample estimation results.

Explanatory Variables	(1)	(2)	(3)	(4)
lnDE	0.0057 (0.58)	0.0334 ** (2.08)	−0.0170 (−1.55)	0.0326 ** (2.01)
rd			−0.0029 (−1.28)	0.0008 (0.38)
lnH			−0.0043 (−0.98)	−0.0038 (−0.95)
lnsagglo			−0.0295 ** (−2.25)	−0.0249 ** (−2.06)
lnfdi			−0.0012 (−0.57)	−0.0009 (−0.50)
lnpgdp			0.0566 *** (4.95)	0.0385 ** (2.28)
cons	−0.3684 *** (−32.31)	−0.4063 *** (-23.41)	−0.8692 *** (−7.62)	−0.7477 *** (−4.40)
*with* $R^{2}$	0.0002	0.1924	0.0152	0.1968
City fixed effect	YES	YES	YES	YES
Time fixed effect	NO	YES	NO	YES
Number of samples	2475	2475	2475	2475

Note: In the table, the t statistic is in parentheses; ** and *** are significant at 5% and 1% levels, respectively.

**Table 5:** Robustness test results.

Explanatory Variables	(1)	(2)
lnDE	0.0686 *** (3.17)	0.0284 * (1.93)
rd	0.0091 *** (4.58)	0.0074 ** (2.55)
lnH	−0.0062 ** (−2.46)	−0.0203 (−1.54)
lnsagglo	−0.0423 *** (−3.95)	0.0168 (0.88)
lnfdi	0.0101 *** (6.45)	−0.0010 (−0.47)
lnpgdp	−0.0187 ** (−2.20)	0.0196 (0.82)
cons	−0.3163 *** (−4.43)	−0.2518 (−0.88)
$R^{2}$	0.1592	0.2928
Kleibergen–Paap rk LM	193.930 [0.0000]	
Kleibergen–Paap rk Wald F	581.9760 {19.93}	

Note: The t statistic is in parentheses, the adjoint probability is in square brackets, and the critical value of the stock Yogo test is in braces. *, **, and *** are significant at 10%, 5%, and 1% levels, respectively.

**Table 6:** Test results of the action mechanism.

Explanatory Variables	(1)	(2)	(3)
lnDE	0.2446 *** (3.61)	0.1380 *** (3.68)	0.0504 *** (3.51)
rd	0.0283 *** (3.27)	−0.0162 *** (−3.38)	0.0018 (1.00)
lnH	0.0263 (1.58)	−0.0042 (−0.46)	−0.0017 (−0.48)
lnsagglo	−0.1455 *** (−2.89)	−0.0009 (−0.03)	0.0010 (0.09)
lnfdi	0.0162 ** (2.06)	−0.0223 *** (−5.13)	0.0011 (0.67)
lnpgdp	0.3106 *** (4.40)	−0.4147 *** (−10.63)	0.1024 *** (6.85)
cons	2.5611 *** (3.61)	5.0619 *** (12.89)	9.1528 *** (60.84)
*with* $R^{2}$	0.6753	0.6068	0.8843
City fixed effect	YES	YES	YES
Time fixed effect	YES	YES	YES
Number of samples	2475	2475	2475

Note: In the table, the t statistic is in parentheses; ** and *** are significant at 5% and 1% levels, respectively.

**Table 7:** Specific classification of cities.

Large Cities	Small and Medium-Sized Cities
Beijing, Tianjin, Shijiazhuang, Tangshan, Qinhuangdao, Handan, Baoding, Zhangjiakou, Hengshui, Taiyuan, Datong, Changzhi, Hohhot, Baotou, Chifeng, Shenyang, Dalian, Fushun, Panjin, Changchun, Jilin, Harbin, Qiqihar, Shanghai, Nanjing, Wuxi, Xuzhou, Changzhou, Suzhou, Nantong, Lianyungang, Huai’an, Yancheng, Yangzhou, Zhenjiang, Taizhou, Suqian, Hangzhou, Ningbo, Wenzhou, Huzhou, Shaoxing, Taizhou, Hefei, Wuhu, Bengbu, Huainan, Huaibei, Fuyang, Suzhou, Lu’an, Bozhou, Fuzhou, Xiamen, Putian, Quanzhou, Longyan, Nanchang, Jiujiang, Ganzhou, Yichun, Fuzhou, Shangrao, Jinan, Qingdao, Zibo, Zaozhuang, Dongying, Yantai, Weifang, Jining, Tai’an, Weihai, Rizhao, Linyi, Dezhou, Liaocheng, Binzhou, Heze, Zhengzhou, Kaifeng, Luoyang, Pingdingshan, Anyang, Xinxiang, Xuchang, Luohe, Nanyang, Shangqiu, Xinyang, Wuhan, Shiyan, Yichang, Ezhou, Jingzhou, Changsha, Zhuzhou, Hengyang, Yueyang, Changde, Yiyang, Yongzhou, Guangzhou, Shenzhen, Zhuhai, Shantou, Foshan, Jiangmen, Zhanjiang, Maoming, Zhaoqing, Huizhou, Yangjiang, Qingyuan, Dongguan, Zhongshan, Chaozhou, Jieyang, Nanning, Liuzhou, Guilin, Qinzhou, Guigang, Yulin, Hezhou, Hechi, Laibin, Haikou, Chongqing, Chengdu, Zigong, Luzhou, Mianyang, Suining, Neijiang, Leshan, Nanchong, Meishan, Yibin, Guang’an, Ziyang, Guiyang, Zunyi, Anshun, Bijie, Kunming, Qujing, Xi’an, Baoji, Hanzhong, Ankang, Lanzhou, Tianshui, Wuwei, Xining, Yinchuan, Urumqi	Xingtai, Chengde, Cangzhou, Langfang, Yangquan, Jincheng, Shuozhou, Jinzhong, Yuncheng, Xinzhou, Linfen, Luliang, Wuhai, Tongliao, Ordos, Hulunbuir, Bayannur, Ulanchap, Benxi, Dandong, Jinzhou, Yingkou, Fuxin, Chaoyang, Huludao, Siping, Liaoyuan, Tonghua, Baishan, Baicheng, Jixi, Hegang, Shuangyashan, Yichun, Jiamusi, Qitaihe, Mudanjiang, Heihe, Suihua, Jiaxing, Jinhua, Quzhou, Zhoushan, Lishui, Maanshan, Tongling, Anqing, Huangshan, Chuzhou, Chizhou, Xuancheng, Sanming, Zhangzhou, Nanping, Ningde, Jingdezhen, Pingxiang, Xinyu, Yingtan, Ji’an, Hebi, Jiaozuo, Puyang, Sanmenxia, Zhoukou, Zhumadian, Huangshi, Jingmen, Xiaogan, Xianning, Suizhou, Xiangtan, Shaoyang, Zhangjiajie, Chenzhou, Huaihua, Loudi, Shaoguan, Meizhou, Shanwei, Heyuan, Yunfu, Wuzhou, Beihai, Fangchenggang, Baise, Chongzuo, Sanya, Panzhihua, Deyang, Guangyuan, Ya’an, Liupanshui, Tongren, Yuxi, Baoshan, Zhaotong, Lijiang, Pu’er, Lincang, Tongchuan, Xianyang, Weinan, Yulin, Shangluo, Jiayuguan, Jinchang, Baiyin, Zhangye, Pingliang, Qingyang, Dingxi, Longnan, Shizuishan, Wuzhong, Guyuan, Zhongwei, Karamay

**Table 8:** Test results of urban scale heterogeneity.

Explanatory Variables	Big Cities	Medium and Small Cities
lnDE	0.0318 * (1.73)	0.0401 (1.38)
rd	−0.0009 (−0.42)	0.0052 (1.23)
lnH	−0.0056 (−1.27)	−0.0044 (−0.63)
lnsagglo	−0.0103 (−0.75)	−0.0501 ** (−2.35)
lnfdi	0.0003 (0.11)	−0.0010 (−0.38)
lnpgdp	0.0762 *** (3.83)	−0.0025 (−0.09)
cons	−1.0852 *** (−5.35)	−0.3970 (−1.39)
*with* $R^{2}$	0.2218	0.2055
City fixed effect	YES	YES
Time fixed effect	YES	YES
Number of samples	1413	1062

Note: In the table, the t statistic is in parentheses; *, **, and *** are significant at 10%, 5%, and 1% levels, respectively.

**Table 9:** Direct and indirect effects of the digital economy on urban environmental quality.

Effect Types	Explanatory Variables	Geographic Distance	Economic Distance	Nested Matrix
Direct effect	lnDE	0.0445 *** (2.68)	0. 0295 * (1.71)	0.0367 ** (2.17)
	rd	−0.0008 (−0.42)	0.0015 (0.82)	0.0002 (0.09)
	lnH	−0.0035 (−0.97)	−0.0047 (−1.29)	−0.0042 (−1.17)
	lnsagglo	−0.0256 ** (−2.28)	−0.0198 * (−1.77)	−0.0228 ** (−2.03)
	lnfdi	−0.0017 (−0.97)	−0.0015 (−0.90)	−0.0013 (−0.73)
	lnpgdp	0.0342 ** (2.14)	0.0358 ** (2.35)	0.0332 ** (2.15)
Indirect effect	lnDE	−0.3419 (−1.45)	0.0449 (1.13)	−0.0572 (−0.47)
	rd	0.0620 ** (2.02)	−0.0133 * (−1.65)	0.0318 (1.63)
	lnH	0.1249 (1.52)	0.0107 (0.62)	−0.0185 (−0.71)
	lnsagglo	0.1475 (0.66)	0.0066 (0.18)	0.0905 (0.74)
	lnfdi	0.0424 (1.23)	−0.0038 (−0.68)	−0.0009 (−0.06)
	lnpgdp	−0.0393 (−0.16)	0.1698 *** (3.78)	0.2241 * (1.75)
W∗lnEQ		0.4091 *** (3.16)	0.1036 *** (2.78)	0. 2590 *** (2.99)
Spatial fixation effect		control	control	control
Time fixed effect		control	control	control

Note: In the table, the Z-statistic value is in parentheses; *, **, and *** are significant at 10%, 5%, and 1%, respectively.

## Data Availability

The data are from the Statistical Yearbook of Chinese Cities, the Statistical Yearbook of China’s Regional Economy, the Digital Inclusive Financial Index of Peking University (2011–2019), the Statistical Yearbook of China, the China Research Data Service Platform (CNDRS), the statistical yearbooks of provinces and cities, Bulletin on Urban National Economic Development and Social Statistics. Carbon emission data are taken from the county carbon dioxide emissions calculated by Chen et al. (2020) [43], summarized at the city level, and the data is as of 2017.
